# Quantitative Detection and Biological Propagation of Scrapie Seeding Activity In Vitro Facilitate Use of Prions as Model Pathogens for Disinfection

**DOI:** 10.1371/journal.pone.0020384

**Published:** 2011-05-27

**Authors:** Sandra Pritzkow, Katja Wagenführ, Martin L. Daus, Susann Boerner, Karin Lemmer, Achim Thomzig, Martin Mielke, Michael Beekes

**Affiliations:** 1 P24, Transmissible Spongiform Encephalopathies, Project Group Immune Defense and Pathogenesis, Robert Koch-Institut, Berlin, Germany; 2 ZBS 2, Highly Pathogenic Microorganisms, Center for Biological Security, Robert Koch-Institut, Berlin, Germany; 3 FG 14, Applied Infection Control and Hospital Hygiene, Department of Infectious Diseases, Robert Koch-Institut, Berlin, Germany; Creighton University, United States of America

## Abstract

Prions are pathogens with an unusually high tolerance to inactivation and constitute a complex challenge to the re-processing of surgical instruments. On the other hand, however, they provide an informative paradigm which has been exploited successfully for the development of novel broad-range disinfectants simultaneously active also against bacteria, viruses and fungi. Here we report on the development of a methodological platform that further facilitates the use of scrapie prions as model pathogens for disinfection. We used specifically adapted serial protein misfolding cyclic amplification (PMCA) for the quantitative detection, on steel wires providing model carriers for decontamination, of 263K scrapie seeding activity converting normal protease-sensitive into abnormal protease-resistant prion protein. Reference steel wires carrying defined amounts of scrapie infectivity were used for assay calibration, while scrapie-contaminated test steel wires were subjected to fifteen different procedures for disinfection that yielded scrapie titre reductions of ≤10^1^- to ≥10^5.5^-fold. As confirmed by titration in hamsters the residual scrapie infectivity on test wires could be reliably deduced for all examined disinfection procedures, from our quantitative seeding activity assay. Furthermore, we found that scrapie seeding activity present in 263K hamster brain homogenate or multiplied by PMCA of scrapie-contaminated steel wires both triggered accumulation of protease-resistant prion protein and was further propagated in a novel cell assay for 263K scrapie prions, i.e., cerebral glial cell cultures from hamsters. The findings from our PMCA- and glial cell culture assays revealed scrapie seeding activity as a biochemically and biologically replicative principle in vitro, with the former being quantitatively linked to prion infectivity detected on steel wires in vivo. When combined, our in vitro assays provide an alternative to titrations of biological scrapie infectivity in animals that substantially facilitates the use of prions as potentially highly indicative test agents in the search for novel broad-range disinfectants.

## Introduction

Prions are the causative agents of transmissible spongiform encephalopthies (TSEs) such as scrapie in sheep and goats, bovine spongiform encephalopathy (BSE) in cattle, chronic wasting disease (CWD) in cervids or Creutzfeldt-Jakob disease (CJD) and its variant form (vCJD) in humans. They are thought to consist essentially of host-encoded prion protein (PrP) with a pathological folding and aggregation structure, referred to as PrP^Sc^ [1], [2] or PrP^TSE^ [3]. Substantial evidence suggests that the replication of prions is mediated by a process of seeded polymerization [4]. In this process PrP^TSE^ particles (that may or may not contain further components or obtain assistance by helper molecules) exert a proteinaceous seeding activity by putatively acting as nuclei which recruit cellular prion protein (PrP^C^) and incorporate it, in a beta-sheet rich amyloid form, into growing aggregates of misfolded PrP. Fragmentation of such aggregates eventually mediates the multiplication of PrP particles with proteinaceous seeding activity, resulting in autocatalytic replication of the pathological protein state. Experimentally, prion-associated seeding activity converting normal protease-sensitive PrP into Proteinase K-resistant prion protein (PrPres) can be monitored *in vitro* by protein misfolding cyclic amplification (PMCA) [5], [6].

Serial PMCA [7], [8] has been established during the past few years as a powerful tool for the ultra-sensitive - yet generally non-quantitative - detection of minute amounts of PrP^TSE^. Chen et al. [9] and Wilham et al. [10] recently described two technical advancements, called quantitative PMCA (qPMCA) and real-time quaking induced conversion assay (RT-QuIC), which showed that the estimation of prion titres and prion seeding activity, respectively, are biochemically feasible *in vitro* with high sensitivity and accuracy.

Prion infectivity can be titrated also biologically *in vitro*, as was exemplified by cell culture assays for the quantitative detection of RML prions in solution or on steel wires used as model carriers for disinfection [11]–[13].

Although the exact chemical composition of prions has not yet been completely elucidated, it is known since long that these pathogens have a high resistance to inactivation (for review see [14], [15]) which resulted in cases of accidental and iatrogenic transmissions of CJD [16]. With their proteinaceous nature, high tolerance against inactivation and pronounced binding affinity to steel surfaces [17], [18] prions provide in many respects a worst case contamination of surgical instruments and medical devices [19], [20] that constitutes both a substantial challenge and informative paradigm for disinfection.

Indeed, prions were recently successfully exploited as model pathogens for the development of novel broad-range disinfectants with simultaneous activity against microbial, viral and TSE agents [21], [22]. In these reports the 263K scrapie hamster model that has been applied for many TSE inactivation studies [14], [15] was used in conjunction with a steel wire assay mimicking the surface of surgical instruments [17], [18], [23]–[25]. For our previous study [22] we devised a three-stage test procedure in which candidate formulations for broad-range disinfection were screened initially for their ability to reduce the burden of 263K scrapie-associated PrP^TSE^ on test steel wires. Formulations found to be effective on PrP^TSE^ in this assay were then tested microbiologically for their activities against bacteria, viruses and fungi, and those showing a pronounced broad-range disinfection activity *in vitro* were finally validated by titrating the decrease of prion infectivity in hamster bioassays.

The identification of a simple yet highly effective formulation for fast broad-range decontamination of surgical instruments from bacteria, viruses, fungi and prions by this approach [22] provided a proof-of-principle for the use of prions as model agents in the search for novel broad-range disinfectants. This prompted us to further refine the monitoring of the activity of disinfectant formulations against prions in terms of speed, throughput, costs and ethical considerations on the protection of animals [26]. Conceptually, prion replication by seeded polymerization would implicate the seeding activity of PrP^TSE^ as the biochemical analogue of biological prion infectivity. Thus, we examined whether it would be possible to assess scrapie infectivity in our steel wire assay without titrations in animals by biochemical measurement and biological detection of 263K scrapie seeding activity *in vitro*. Specifically adapted quantitative serial PMCA and a novel glial cell culture assay were tested for this purpose.

## Results

### Quantitative PMCA for measuring the proteinaceous seeding activity of 263K scrapie prions in vitro

When we developed a protocol for quantitative PMCA of prion contaminated steel wires we took advantage of previous findings in our laboratory indicating that the presence of glass beads in reaction batches may substantially increase both the robustness and sensitivity of PMCA. However, the size and amount of beads had to be adequately adjusted for this purpose. Similar findings were described recently by Gonzales-Montalban et al. [27]. Furthermore, as a safeguard to PMCA specificity we implemented several different precautionary measures aiming at the prevention of cross-contamination.

**Figure 1:**
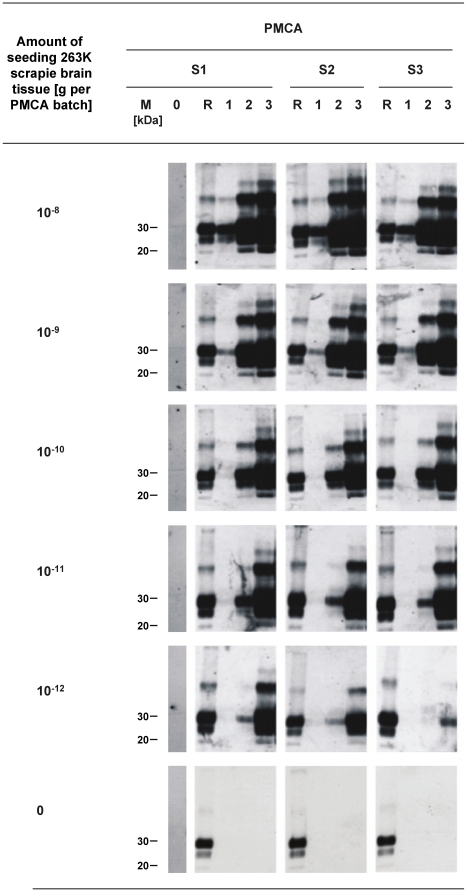
Reproducibility, sensitivity and specificity of PMCA optimized for the quantitative determination of scrapie seeding activity. Western blot detection of PrPres, the proteinase K–resistant core of misfolded PrP, after PMCA seeded with the indicated amounts of 263K scrapie brain tissue. Samples were run in triplicates (S1–S3). Lane M, markers indicating the typical molecular mass of PrPres in the range of ∼30 to ∼20 kDa. Lane 0 represents 4.2 µl-aliquots from PMCA batches sampled prior to PMCA. Lane R, PrPres reference standard: PK-digested brain homogenate from scrapie hamsters corresponding to 5×10^−7^ g brain tissue. Lanes 1–3 represent 4.2 µl-aliquots from PMCA batches sampled after 1, 2 and 3 rounds of amplification.

Figure 1 shows the sensitivity and specificity of the detection of scrapie seeding activity, as well as the consistency of PrPres amplification triggered by 263K scrapie prions, for PMCA samples that were processed, in triplicate, according to our modified PMCA protocol. No PrP^TSE^ of the seeding material was detected by Western blotting prior to PMCA in reaction batches containing 10^−8^ g/150 µl or lower concentrations of homogenized 263K scrapie brain tissue. This is consistent with the previously established sensitivity of our Western blot assay which allows the detection of PrP^TSE^ in the equivalent of 2×10^−8^ g or higher amounts of homogenized brain tissue from terminally ill scrapie hamsters [28]. In contrast, seeding activity could be detected in all PMCA batches containing 10^−12^ g or higher amounts of 263K scrapie brain tissue. When all safety measures against cross-contamination had been in place unseeded samples did not produce PrPres staining after 1, 2 or 3 rounds of PMCA (Figure 1, bottom row).

However, we observed that the efficacy, though not the consistency of PrPres amplification detected for identically prepared sets of triplicate samples depended to some degree on both the particular sonicator used for PMCA and the individual run of sample processing at different times in the same sonicator (not shown). This variation of protein misfolding efficacy found to be intrinsically associated with PMCA in our hands prompted us to perform an internal assay calibration for the quantitative determination of scrapie seeding activity. Accordingly, unknown samples to be tested for their seeding activity were subjected to PMCA always together with defined reference samples and processed in one sample set at the same time in the same sonicator.

**Figure 2:**
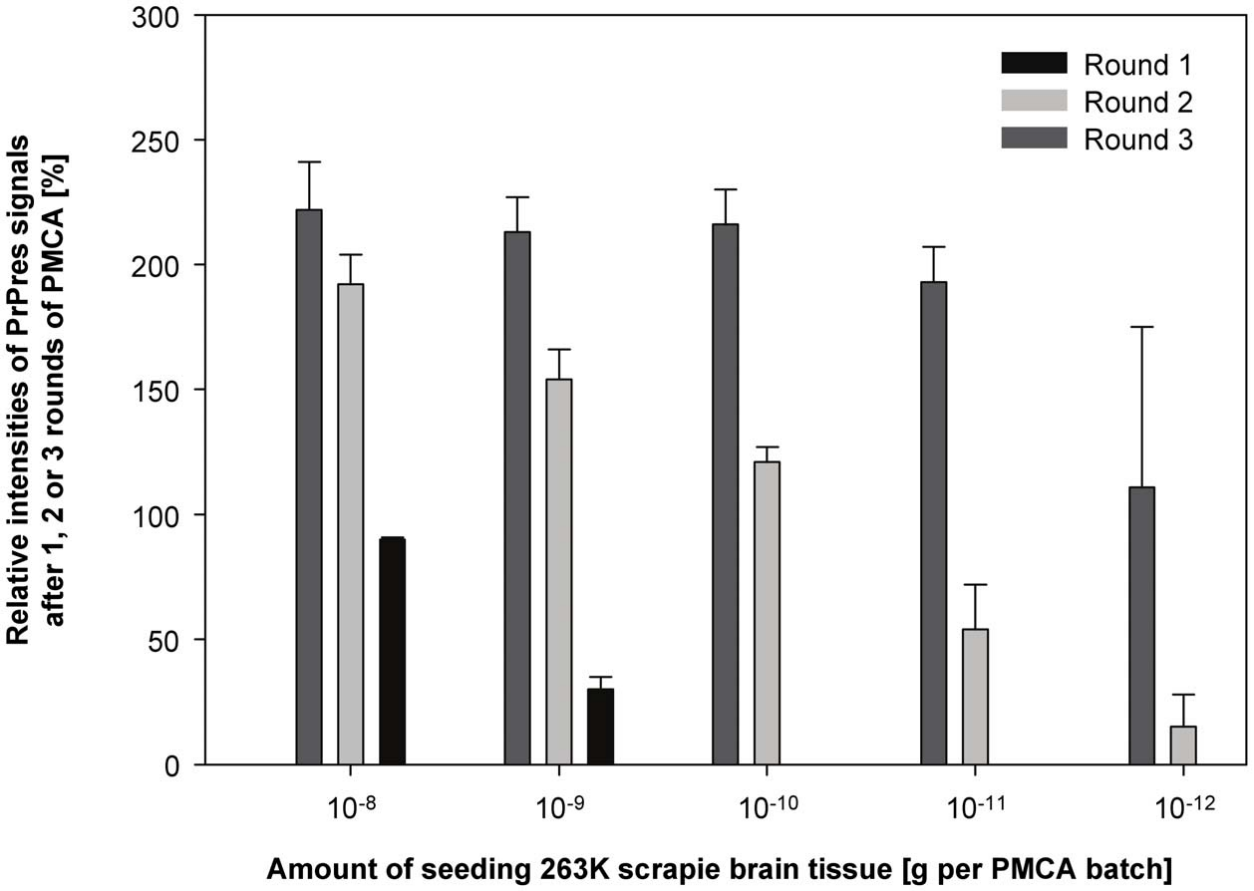
Quantitative correlation between the amount of seeding 263K scrapie brain tissue, number of PMCA rounds and level of PrPres amplification. Densitometric analysis of the Western blots shown in Figure 1. Relative staining intensities of PrPres found after 1, 2 and 3 rounds of PMCA seeded with the indicated amounts of 263K scrapie brain tissue are expressed as a percentage of the staining intensity of the reference standard. Upper parts of symmetrical error bars are reproduced in order to indicate the variation (i. e. the range) of PrPres staining observed for triplicate samples.

Under the experimental conditions of our PMCA protocol, the amount of PrPres generated after different rounds of PMCA directly reflected the levels of seeding activity present in examined samples. When the Western blot results shown in Figure 1 were analysed densitometrically this confirmed a consistent quantitative correlation between the amount of seeding material, the number of PMCA rounds, and the staining intensity of generated PrPres (Figure 2). In the following we used, and validated, this PMCA protocol for the measurement of the scrapie seeding activity on steel wires in a carrier assay for disinfection.

### Quantification of proteinaceous seeding activity on prion-contaminated steel wires in a carrier assay for disinfection

**Figure 3:**
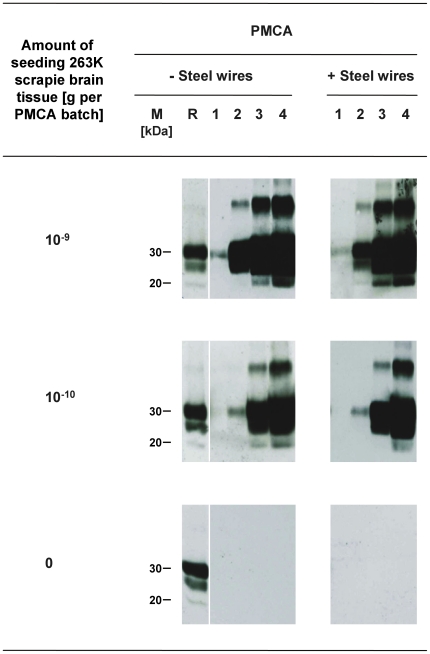
Influence of steel wires on the efficacy of PMCA. Western blot detection of PrPres, the proteinase K–resistant core of misfolded PrP, after PMCA seeded with the indicated amounts of 263K scrapie brain tissue and performed in the absence (−) or presence (+) of untreated steel wires. Lane M, markers indicating the typical molecular mass of PrPres in the range of ∼30 to ∼20 kDa. Lane R, PrPres reference standard: PK-digested brain homogenate from scrapie hamsters corresponding to 1×10^−6^ g brain tissue. Numbered lanes 1–4 represent 4.2 µl-aliquots from PMCA batches sampled after 1, 2, 3 or 4 rounds of amplification.

Before addressing the quantification of proteinaceous seeding activity on steel wires, we examined whether the presence of such wires in reaction batches influenced the efficacy of PMCA. As shown in Figure 3 the addition of untreated steel wires to normal hamster brain homogenate (NBH) spiked with 10^−9^ or 10^−10^ g of 263K scrapie brain tissue had no significant impact on PrPres amplification. Furthermore, no PrPres signals could be detected after 1, 2, 3 or 4 PMCA rounds with unseeded NBH containing steel wires. Hence, we observed no substantial effects of steel wires on PMCA: Such wires did neither boost specific or unspecific PrPres amplification nor did they cause relevant inhibitory effects in our assay.

Next, we applied our PMCA procedure to prion-contaminated steel wires for an assessment of scrapie seeding activities in a carrier assay for disinfection. Test-, reference- and negative control steel wires were included into the analysis. Scrapie-contaminated test steel wires were subjected to various procedures for disinfection, while reference steel wires carried defined amounts of scrapie agent for assay calibration. Negative control steel wires were contaminated with 10^−1^-diluted NBH, only. Different sample sets each containing i) test wires that had been exposed to procedures for disinfection, ii) reference wires for assay calibration and iii) negative control wires were assorted, and the samples belonging to one sample set were subjected together, i. e. at the same time and in the same sonicator, to PMCA.

**Figure 4:**
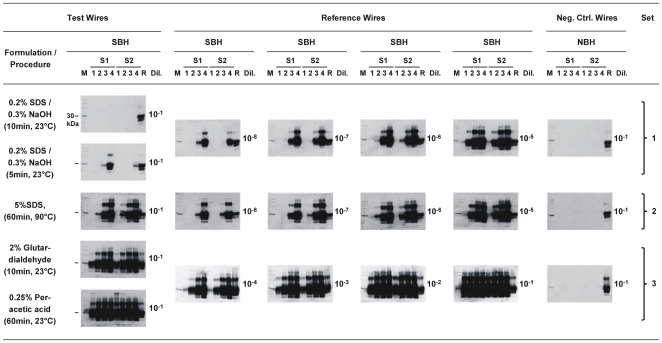
Effect of disinfectants on the proteinaceous seeding activity of prion-contaminated steel wires. Western blot detection of PrPres, the proteinase K–resistant core of misfolded PrP, after PMCA seeded with duplicate samples (S1, S2) of test-, reference- and negative control (neg. ctrl.) steel wires that had been contaminated with the indicated dilutions of 263K scrapie- or normal hamster brain homogenate (SBH, NBH). Test wires were exposed to the specified formulations or procedures for disinfection and subsequently subjected to PMCA together with reference and control wires (Note: 2% glutardialdehyde was not neutralized). Results from three independently processed PMCA sample sets are shown. Each sample set consisted of i) test wires that had been exposed to procedures for disinfection (Set 1: 0.2% SDS/0.3% NaOH applied for 10 min or 5 min at 23°C; Set 2: 5% SDS applied for 60 min at 90°C; Set 3: 2% glutardialdehyde applied for 10 min at 23°C and 0.25% peracetic acid applied for 60 min at 23°C), ii) reference wires contaminated with 10^−5^- to 10^−8^-diluted SBH (Sets 1 and 2) or 10^−1^- to 10^−4^-diluted SBH (Set 3) for assay calibration in the range of the residual seeding activity on test wires, and iii) negative control wires contaminated with 10^−1^-diluted NBH (Sets 1, 2 and 3). The test-, reference- and negative control wire samples belonging to one sample set were subjected together, i. e. at the same time and in the same sonicator, to PMCA. Lane M, molecular mass marker; the prominent band (indicated by bars in the first column of blots) corresponds to 30 kDa. Lane R, PrPres reference standard: PK-digested brain homogenate from scrapie hamsters corresponding to 1×10^−6^ g brain tissue. Numbered lanes 1–4 represent 4.2 µl-aliquots from PMCA batches sampled after 1, 2, 3 or 4 rounds of amplification. Lane “Dil.”, dilution of 263K SBH (in 10% NBH) or NBH (in buffer) with which wires had been contaminated prior to PMCA.

Figure 4 shows the amplification of PrPres after 1, 2, 3 and 4 rounds of PMCA seeded with duplicates (S1, S2) of test-, reference- and negative control (neg. ctrl.) steel wires that had been contaminated with the indicated dilutions of scrapie hamster brain homogenate (SBH) or NBH. As previously observed for PMCA without steel wires, the efficacy of PrPres amplification depended to some degree on the very sonicator (or the individual run of sample set processing at different times in the same sonicator) for sample sets 1–3. However, duplicate test- and reference samples processed together at the same time in the same sonicator produced consistent PrPres amplification. With negative control wires no PrPres amplification was detected.

No PrPres could be detected after 1, 2, 3 or 4 rounds of PMCA following exposure of test wires (that had been coated with 10^−1^-diluted SBH) to a formulation of 0.2% SDS (sodium dodecylsulfate) and 0.3% NaOH for 10 min at 23°C, whereas reference wires contaminated with 10^−8^-diluted SBH produced detectable PrPres amplification after 3 (S1) and 4 rounds (S1 and S2) of PMCA (Figure 4, sample set 1). This indicated that after treatment in 0.2% SDS/0.3% NaOH for 10 min at 23°C any residual seeding activity on these test wires (SA_TW_[0.2% SDS/0.3% NaOH, 10 min, 23°C]), if present at all, was lower than the seeding activity of reference wires contaminated with 10^−8^-diluted SBH (SA_RW_[10<sup>−8</sup>]). Increasingly stronger residual seeding activities were revealed in the Western blots of Figure 4 after exposure of test wires to 0.2% SDS/0.3% NaOH for 5 min at 23°C, 5% SDS (pH 7.6) for 60 min at 90°C, 2% non-neutralised glutardialdehyde (pH 4.6) for 10 min at 23°C, or 0.25% peracetic acid for 60 min at 23°C.

**Figure 5:**
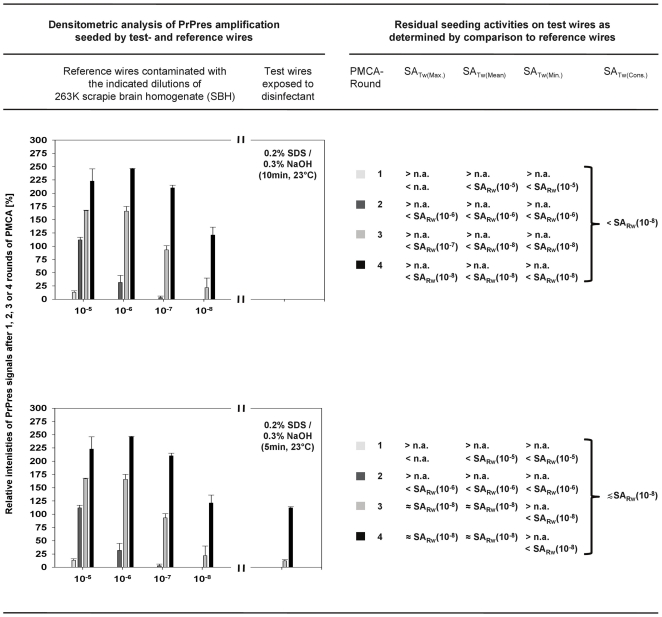
Quantification of residual proteinaceous seeding activities on prion-contaminated steel wires after exposure to a mixture of 0.2% SDS and 0.3% NaOH. (Left panel) Densitometric analysis of PrPres amplification detected for test- and reference wires in sample set 1 of Figure 4. The horizontal axis of plots represents reference wires contaminated with the indicated dilutions of SBH (left to axis break), or test wires exposed to 0.2% SDS/0.3% NaOH for the indicated reaction times (right to axis break). The vertical axis of plots indicates the relative staining intensities of PrPres signals found for reference or test wires after 1, 2, 3 and 4 rounds of PMCA expressed as a percentage of the staining intensity of the reference standard (i. e. PK-digested brain homogenate from scrapie hamsters corresponding to 1×10^−6^ g brain tissue). The color coding of bars representing the first, second, third and fourth round of PMCA is defined in the column “PMCA-Round” of the right panel. Upper parts of symmetrical error bars are reproduced in order to indicate the variation (i. e. the range) of results for duplicate reference samples and individual test samples after three independently performed densitometric measurements of Western blot signals. (Right panel) Classification of residual seeding activities of test wires in relation to the seeding activities of reference wires for 1, 2, 3 and 4 rounds of PMCA, and deduction of consolidated values of residual seeding activities on test wires (assessments refer to the reaction times of 0.2% SDS/0.3% NaOH specified in the plots on the left hand side). Explanation of abbreviations: SA_TW[Max.]_, SA_TW[Mean]_, SA_TW[Min.]_ and SA_TW[Cons.]_; maximum-, mean-, minimum and consolidated values of the seeding actvity of test wires. N.a., no assessment. SA_RW_(10^−5^) - SA_RW_(10^−8^) designate seeding activities of reference wires contaminated with 10^−5^- to 10^−8^-diluted SBH.

**Figure 6:**
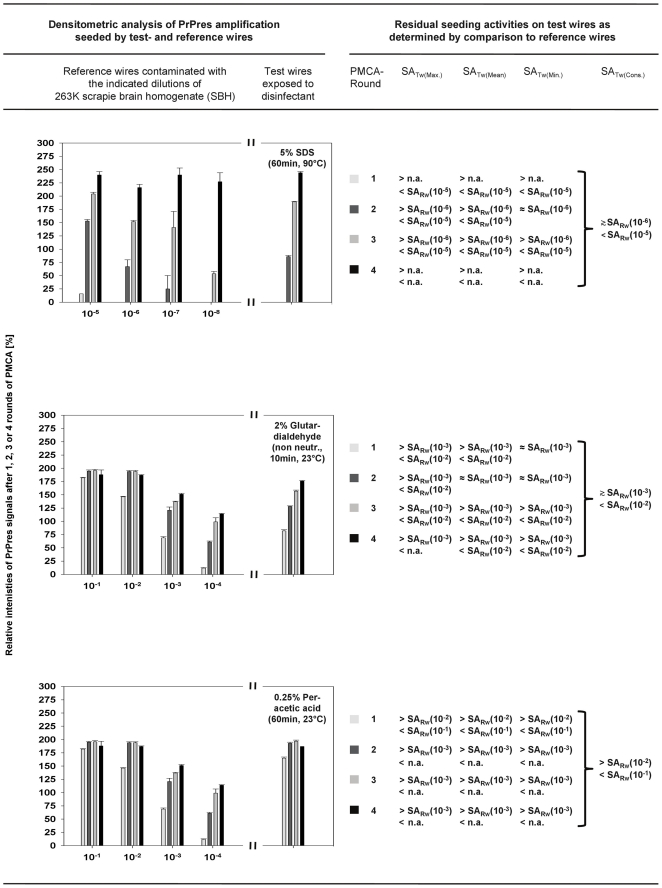
Quantification of residual proteinaceous seeding activities on prion-contaminated steel wires after exposure to SDS, glutardialdehyde or peracetic acid. (Left panel) Densitometric analysis of PrPres amplification detected for test- and reference wires in sample sets 2 and 3 of Figure 4. The horizontal axis of plots represents reference wires contaminated with the indicated dilutions of SBH (left to axis break), or test wires exposed to the specified disinfectant (right to axis break). Vertical axis, color coding of bars and error bars as described for Figure 5. (Right panel) Classification of residual seeding activities on test wires exposed to the disinfectants specified in the plots on the left hand side (the analyses were performed similarly to Figure 5). Abbreviations: As in Figure 5, with SA_RW_(10^−1^) - SA_RW_(10^−8^) designating seeding activities of reference wires contaminated with 10^−1^- to 10^−8^-diluted SBH.

All PMCA results were also analysed by densitometry. The relative staining intensities of PrPres signals found for test- and reference wires after 1, 2, 3 and 4 rounds of PMCA were densitometrically determined as a percentage of the staining intensities of PrPres reference standards and plotted as exemplified in Figures 5 and 6 (left columns) for the Western blots of sample set 1 or samples sets 2 and 3 of Figure 4, respectively.

The densitometric analysis confirmed that any residual seeding activity on test wires exposed to 0.2% SDS/0.3% NaOH for 10 min at 23°C was lower than the seeding activity on reference wires coated with 10^−8^-diluted SBH (i. e. SA_TW_[0.2% SDS/0.3% NaOH, 10 min, 23°C] < SA_RW_[10<sup>−8</sup>]; Figure 5, first panel), while after the same treatment for 5 min the residual seeding activity on the test wires was ≲ SA_RW_(10^−8^) (Figure 5, second panel). After treatment in 5% SDS (pH 7.6) for 60 min at 90°C, 2% non-neutralised glutardialdehyde for 10 min at 23°C or 0.25% peracetic acid for 60 min at 23°C the residual seeding activities on test wires were found to be ≳ SA_RW_(10^−6^) to < SA_RW_(10^−5^) (Figure 6, first panel), ≳ SA_RW_(10^−3^) to < SA_RW_(10^−2^) (Figure 6, second panel) and > SA_RW_(10^−2^) to < SA_RW_(10^−1^) (Figure 6, third panel), respectively.

In addition to these treatments a further ten different formulations or procedures with efficacies for disinfection known to range from very high to very low were examined for their effects on the scrapie seeding activity on test wires. For each disinfectant formulation/procedure the seeding activity assay was carried out in quadruplicate and produced consistent results. The consolidated data from our seeding activity assay are summarized in Table 1. Minor variations between quadruplicate seeding activity measurements were observed for 0.2% SDS/0.3% NaOH/20% n-propanol (10 min, 23°C), 0.5% alkaline cleaner (5 min, 55°C) and steam sterilization (5 min, 134°C). However, these variations were either consistent with, or even paralleled by bioassay findings on test wires (Table 1).

**Table 1:** Seeding activity assay and bioassay of test steel wires used as model carriers for prion disinfection.

				Seeding activity assay of test wires			Bioassay of test wires
Formulation/	Concen-	Time	Tempera-	Residual seeding activity on test wires	Estimated residual infectivity	Estimated	Residual infectivity	Reduction of
Procedure	tration	[min]	ture [°C]	(as compared to the seeding activity	per wire	reduction of	per wire	infectivity
				on reference wires)	[LD_50i.c.imp_]	infectivity [logs]	[LD_50i.c.imp_]	[logs]#
Sodium hydroxide	1.0 M	60	23	< SA_RW_(10^−8^)	<3×10^−2^	>7	UD†	≥5.5†
Sodium hypochlorite	2.5%	60	23	≲ SA_RW_(10^−8^)	≲3×10^−2^	≳ 7	UD†	≥5.5†
GdnSCN	4.0 M	10	23	≲ SA_RW_(10^−8^)	≲3×10^−2^	≳ 7	UD¶	≥5.5¶
SDS/NaOH	0.2%/0.3%	10	23	< SA_RW_(10^−8^)	<3×10^−2^	>7	UD†	≥5.5†
		5	23	≲ SA_RW_(10^−8^)	≲3×10^−2^	≳ 7	UD†	≥5.5†
SDS/NaOH/	0.2%/0.3%	10	23	≲ SA_RW_(10^−8^)	≲3×10^−2^	≳ 7	UD‡	≥5.5‡
n-Propanol	20%			> SA_RW_(10^−8^) to ≲ SA_RW_(10^−7^)	>3×10^−2^ to ≲3×10^−1^	>6 to ≲ 7		
Alkaline cleaner	1.0%	60	23	< SA_RW_(10^−8^)	<3×10^−2^	>7	UD†	≥5.5†
	0.5%	10	55	> SA_RW_(10^−7^) to ≲ SA_RW_(10^−6^)	>3×10^−1^ to ≲3×10^0^	≳ 5 to <6	>0 to ≤3×10^0^ †	≥5 to ≤5.5†
	0.5%	5	55	> SA_RW_(10^−7^) to ≲ SA_RW_(10^−6^)	>3×10^−1^ to ≲3×10^0^	≳ 5 to <6	>0 to ≤3×10^0^ †	≥5 to ≤5.5†
				> SA_RW_(10^−6^) to ≲ SA_RW_(10^−5^)	>3×10^0^ to ≲3×10^1^	≳ 4 to <5	>3×10^0^ to ≤3×10^1^ †	≥4 to <5†
Steam sterilization		5	134	≈ SA_RW_(10^−6^)	≈3×10^0^	≈ 5	>0 to <3×10^0^ †	>5 to <5.5†
				≳ SA_RW_(10^−6^) to < SA_RW_(10^−5^)	≳3×10^0^ to <3×10^1^	>4 to ≲ 5		
SDS (pH 7.6)	5%	60	90	≳ SA_RW_(10^−6^) to < SA_RW_(10^−5^)	≳3×10^0^ to <3×10^1^	>4 to ≲ 5	>3×10^0^ to ≤3×10^1^ †	≥4 to <5†
Glutardialdehyde	2%	10	23	> SA_RW_(10^−4^) to ≲ SA_RW_(10^−3^)	>3×10^2^ to ≲3×10^3^	≳2 to <3	≈3×10^3¶^	≈ 2¶
(neutral., pH 7.0)								
Glutardialdehyde	2%	10	23	≳ SA_RW_(10^−3^) to < SA_RW_(10^−2^)	≳3×10^3^ to <3×10^4^	>1 to ≲ 2	>3×10^3^ to <3×10^4¶^	>1 to <2¶
non-neutral., pH 4.6)								
Cidex OPA	0.55%	10	23	> SA_RW_(10^−3^) to < SA_RW_(10^−2^)	>3×10^3^ to <3×10^4^	>1 to <2	>3×10^3^ to <3×10^4¶^	>1 to <2¶
Peracetic Acid	0.25%	60	23	> SA_RW_(10^−2^) to < SA_RW_(10^−1^)	>3×10^4^ to <3×10^5^	>0 to <1	>3×10^4^ to ≤3×10^5^ †	≥0 to <1†

Test wires contaminated with 10^−1^-diluted 263K scrapie brain homogenate carried an initial infectivity load of approximately 3×10^5^ LD_50i.c.imp_/wire, and reference wires contaminated with 10^−1^-, 10^−2^-, 10^−3^-, 10^−4^, 10^−5^-, 10^−6^, 10^−7^- and 10^−8^-diluted 263K scrapie brain homogenate carried 3×10^5^-, 3×10^4^-, 3×10^3^-, 3×10^2^-, 3×10^1^-, 3×10^0^-, 3×10^−1^- or 3×10^−2^ LD_50i.c.imp_/wire, respectively [29]. Reduction of prion infectivity on test wires is expressed in log_10_ units (logs). Explanation of abbreviations: neutrl. – neutralized; UD - undetectable. Explanation of symbols:

# Test limit of bioassay for titre reduction: 5.5 logs [29];

†, ‡ Previously published bioassay results ([29] ^†^ [22] ^‡^).

¶ Bioassay results from this work (see Table S1 and Table S2).

### In vitro assessment and bioassay validation of prion infectivity on test wires

Test wires similarly processed for decontamination as those examined in our seeding activity assay had been previously, or were in this study, subjected to hamster bioassays for a biological titration of infectivity. The results from the bioassays performed in this study are summarized in the Supporting Information of Table S1 and Table S2, while bioassay results from previous titrations have been published by Lemmer et al. [29] and Beekes et al. [22] (see footnote of Table 1). In addition, the infectivity titres of reference steel wires identical to those coated with serially 10^−1^- to 10^−8^-diluted SBH used in this report had been previously determined as well [29], and could now be applied as conversion factors for a tentative translation of the seeding activities detected on test wires (Table 1, fifth column) into titre estimates of biological prion infectivity (Table 1, sixth column). With this experimental design, we were able to validate our *in vitro* assessments of prion infectivity on test wires by directly comparing estimated titres concluded from the seeding activity assay (Table 1, sixth column) with actual infectivity levels detected in reporter animals (Table 1, eighth column).

The seeding activity assay estimates for test wires exposed to 0.2% SDS/0.3% NaOH for 10 min at 23°C suggested a titre reduction factor (RF) of >7 log_10_ units (logs; Table 1, seventh column). Consistent with this *in vitro* assessment no residual infectivity was detected by bioassay on similarly treated test wires. The bioassay indicated for this treatment an infectivity reduction factor of greater or equal 5.5 logs (Tab 1, ninth column). This was the maximum reduction detectable by bioassay since the initial load of infectivity on test wires contaminated with 10^−1^-diluted SBH was 3×10^5^ 50% lethal doses of 263K scrapie infectivity following intracerebral implantation (LD_50i.c.imp_) per wire [29]. Decontamination with 0.2% SDS/0.3% NaOH for 5 min at 23°C, 5% SDS (pH 7.6) for 10 min at 90°C, 2% non-neutralised glutardialdehyde for 10 min at 23°C or 0.25% peracetic acid for 60 min at 23°C produced residual seeding activities on test wires that were indicative of titre reductions in the range of ≳ 7, >4 to ≲ 5 logs, >1 to ≲ 2 logs and >0 to <1 log, respectively.

As outlined in Table 1 we tested 15 different procedures or formulations for disinfection that yielded estimated reductions of 263K scrapie titres on wires ranging from smaller than 1 log to ≥5.5 logs. We found that the residual infectivities and titre reduction factors on processed test wires suggested by the seeding activity assay consistently matched the findings from bioassays.

### Glial cell culture assay for scrapie seeding activity

In order to qualitatively test, and verify, PrP seeding as a biologically active and replicative principle without experiments in animals, we established a cell culture assay using cerebral glial cells from hamsters. Examination by light microscopy and immunocytochemistry revealed that our cerebral glial cell cultures contained astrocytes (which were identified based on their morphological appearance and by immunolabelling of glial fibrillary acidic protein, not shown) as well as other non-neuronal cells (that were not specifically immunolabelled but morphologically resembled oligodendrocytes and microglia, not shown). The level of PrP^C^ expression regularly found in our glial cell cultures at 40 days post initial exposure (DPE) to NBH, i. e. at a time point when cells were usually harvested for the analysis of PrPres accumulation after challenge with 263K scrapie brain homogenate, is shown in Figure S1 of the Supporting Information. Similar levels of PrP^C^ expression were also found at 60 DPE (not shown). Using these cell cultures for assaying 263K scrapie seeding activity the following results were obtained in experiments carried out in duplicate or a higher number of independent runs.

**Figure 7:**
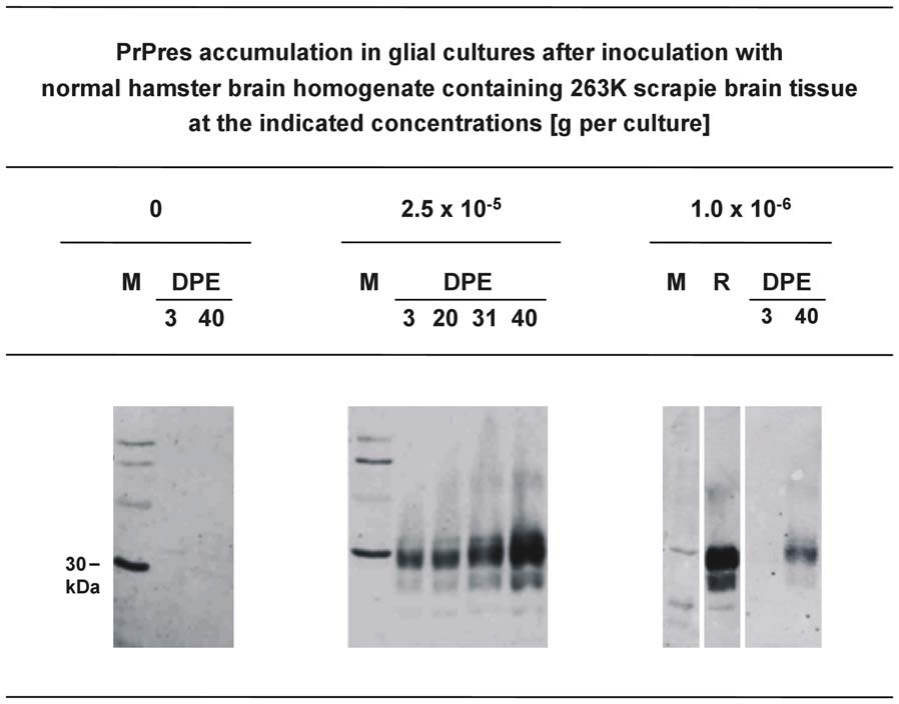
PrPres-accumulation in glial cell cultures exposed to scrapie brain homogenate. Western blot detection of PrPres, the proteinase K–resistant core of misfolded PrP, at the indicated days post initial exposure (DPE) in glial cell cultures from hamsters inoculated with NBH containing 0, 2.5×10^−5^, or 1.0×10^−6^ g 263 K scrapie brain tissue. Lanes DPE represent 3.8 µl-aliquots from resuspended cell culture pellets harvested at the indicated days post initial exposure. Lane M, molecular mass marker; the prominent band (indicated by bar) corresponds to 30 kDa. Lane R, PrPres reference standard: PK-digested brain homogenate from scrapie hamsters corresponding to 5×10^−7^ g brain tissue.

We found that our cultures of cerebral glial cells isolated from newborn Syrian hamsters showed a consistent accumulation of PrPres upon exposure to 263K scrapie brain homogenate when the cells had been adequately dispersed and adjusted to an optimized concentration before inoculation. The cultures were exposed for 3 days to NBH spiked with 2.5×10^−5^ g of scrapie brain tissue, washed, and incubated until 40 DPE. Western blotting of cells harvested at different time-points after inoculation revealed increased levels of PrPres at 31 and 40 DPE as compared to the PrPres staining found at 3 DPE (which probably originated from residual inoculum that could not be washed off the cells; Figure 7, blot in the middle). PrPres accumulation was also observed at 40 DPE after exposure of cells to inoculum that contained 1×10^−6^ g of scrapie brain tissue per culture (Figure 7, blot on the right-hand side), but not consistently when cultures were exposed to 1×10^−7^ g of scrapie brain tissue (not shown). No PrPres could be detected at 3 or 40 DPE in extracts from glial cell cultures similarly exposed to NBH only (Figure 7, blot on the left hand side).

**Figure 8:**
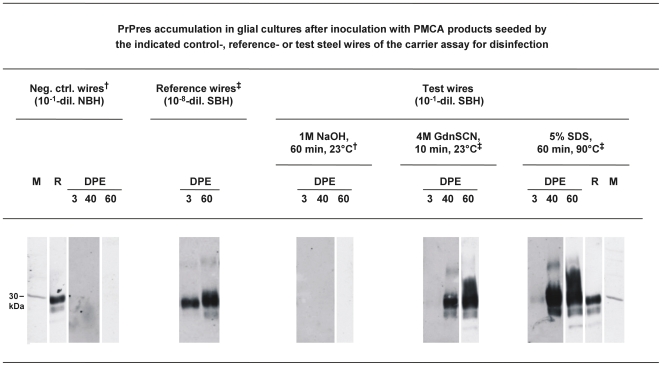
PrPres-accumulation in glial cell cultures exposed to PMCA products. Western blot detection of PrPres at the indicated DPE in glial cell cultures from hamsters inoculated with PMCA products seeded by control-, reference and test steel wires of the carrier assay for disinfection and harvested after the 4^th^ round of amplification. Negative control (neg. ctrl) wires, reference wires and test wires had been contaminated with 10^−1^-diluted (dil.) NBH, 10^−8^-diluted SBH and 10^−1^-diluted SBH, respectively, and test wires were subjected to the indicated disinfection procedures prior to PMCA. PMCA products from negative control wires and test wires exposed to 1M NaOH were PrPres-negative (^†^, see Figure 3 for negative control wires [set 1, S1, 4^th^ round]; otherwise not shown), while PMCA products from reference wires and test wires exposed to 4M GdnSCN or 5% SDS were positive for PrPres (^‡^, see Figure 3 for reference wires [set 2, S1, 4^th^ round] or for test wires incubated in 5% SDS [set 2, S2, 4^th^ round]; otherwise not shown). Lanes DPE represent 3.8 µl-aliquots from resuspended cell culture pellets harvested at the indicated days post initial exposure. Lane M, molecular mass marker; the prominent band (indicated by bar) corresponds to 30 kDa. Lane R, PrPres reference standard: PK-digested brain homogenate from scrapie hamsters corresponding to 5×10^−7^ g brain tissue.

Next, we exposed glial cell cultures for 3 days to PMCA products seeded by reference steel wires coated with 10^−8^-diluted SBH, or by test steel wires exposed to different disinfectant formulations. As shown in Figure 8 the PMCA product seeded by reference steel wires, and the PMCA products seeded by test steel wires that had been treated in 4 M GdnSCN (10 min, 23°C) or 5% SDS (pH 7.6, 60 min, 90°C), triggered PrPres accumulation in the glial cell cultures (Figure 8, second, fourth and fifth blot from the left hand side). This was not observed with PMCA products derived from test steel wires exposed to 1 M NaOH (60 min, 23°C; Figure 8, third blot from the left hand side). Also, control samples from similar PMCA reactions that were mock-seeded by steel wires contaminated with 10^−1^-diluted NBH did not produce detectable PrPres signals in glial cell cultures at 3, 40 or 60 DPE (Figure 8, first blot from the left hand side). The molecular identity of accumulated PrPres in glial cell cultures was confirmed by using an alternative antibody for PrPres labelling (i. e. anti-PrP antibody ICSM-18 [D-Gen, UK] instead of anti-PrP antibody 3F4), as well as by enzymatic deglycosylation [28]. Western blotting with the antibody ICSM-18 revealed bands in the molecular mass range of diglycosylated, monoglycosylated and occasionally ungycosylated PrPres (not shown). Furthermore, after treatment with N-glycosidase F (PNGase F) and Western blot staining with the antibody 3F4 the putative PrP bands shown in Figure 7 displayed a uniform shift to approximately 19 kDa, the molecular mass expected for the unglycosylated form of hamster PrPres (not shown). These findings confirmed the identity of the immunolabelled material in our glial cell cultures as PrPres.

The preparation of primary hamster glial cell cultures and their inoculation with 263K scrapie brain homogenate or PMCA products has been carried out, partly by different operators, in our laboratory in more than 20 independently performed experiments so far. Glial cell cultures consistently accumulated in replicate tests PrPres after exposure to these inocula as exemplified in Figures 7 and 8, indicating that they constantly provided the same quality for prion susceptibility.

**Figure 9:**
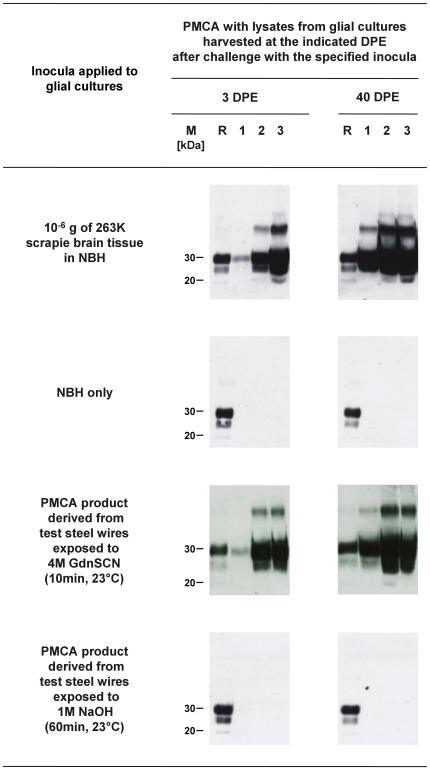
Propagation of seeding activity in glial cell cultures exposed to scrapie brain homogenate or PMCA products. Western blot detection of PrPres, the proteinase K–resistant core of misfolded PrP, after PMCA seeded with lysate extracts from hamster glial cell cultures that had been harvested at 3 or 40 days post initial exposure (DPE) to 263K scrapie brain tissue, normal hamster brain homogenate (NBH), or PMCA products derived from test steel wires treated with 4M GdnSCN (10 min, 23°C) or 1 M NaOH (60 min, 23°C) in the carrier assay for disinfection. Note: Lysate extracts from 3- and 40 DPE glial cell cultures exposed to the PMCA product from test steel wires treated with 4M GdnSCN (10 min, 23°C) were diluted 1:25 for PMCA seeding in this experiment. Lane M, markers indicating the typical molecular mass of PrPres in the range of ∼30 to ∼20 kDa. Lane R, PrPres reference standard: PK-digested brain homogenate from scrapie hamsters corresponding to 5×10^−7^ g brain tissue. Numbered lanes 1–3 represent 4.2 µl-aliquots from PMCA batches sampled after 1, 2 and 3 rounds of amplification.

Furthermore, we validated the biological propagation of PrP seeding in our cell culture assay by testing whether the newly formed PrPres, again, is associated with proteinaceous seeding activity in PMCA. Lysates from glial cell cultures inoculated with 10^−6^ g of homogenized 263K scrapie brain tissue in NBH, or with a PrPres-positive PMCA product derived from test steel wires that had been exposed to 4 M GdnSCN (10 min, 23°C), showed both accumulation of PrPres (Figure 7, blot on the right hand side; Figure 8, fourth blot from the left hand side) and increased seeding activity (Figure 9, first and third row) at 40 DPE as compared to 3 DPE. In contrast, PrPres-negative lysates from glial cell cultures exposed to inocula not containing detectable amounts of PrPres (i. e. NBH [Figure 8, blot on the left hand side], or a PMCA product derived from test steel wires treated in 1 M NaOH for 60 min at 23°C [Figure 8, third blot from the left hand side] did not show any detectable seeding activity in PMCA (Figure 9, second and fourth row of blots).

Thus, scrapie brain tissue as well as PMCA products derived from seeds such as prion contaminated steel wires specifically triggered both accumulation of PK-resistant PrP and amplification of proteinaceous seeding activity in our cell assay. This provided a direct *in vitro* demonstration of PrP seeding as a biologically active and replicative principle, since PrP seeding had been transmitted to and propagated by our glial cell cultures.

## Discussion

Prions have a particularly high tolerance to inactivation [14], [15]. Therefore, they constitute a complex challenge to the safe maintenance of re-usable surgical instruments and medical devices [30]. However, as recently shown, this challenge can be turned into benefit when prions are exploited as an informative paradigm for the development of novel disinfectants that are simultaneously active against bacteria, viruses as well as fungi [21], [22]. In order to further facilitate the use of prions as model agents in the search for novel broad-range disinfectants we established an experimental platform for the sensitive quantitative measurement and biological detection of scrapie seeding activity *in vitro*.

In our study scrapie seeding activity on prion-contaminated steel wires processed for decontamination was quantified by specifically adapted PMCA. PMCA has been used previously by other groups as a rapid test for the assessment of prion inactivation [31], [32], however, not in a quantitative way. PMCA needs to be highly standardized and robust in terms of a consistent and objectively quantifiable PrPres amplification if to be used for a quantification of the proteinaceous seeding activity of prions. Only recently it was reported that conducting PMCA in the presence of Teflon beads significantly improves the yield, rate and robustness of PrP conversion seeded by 263K scrapie prions [27]. When we developed our protocol for quantitative PMCA we observed a similar effect after the addition of glass beads, but not after the addition of steel wires to PMCA reaction batches. Although the exact mechanistic explanation has not yet been established for these phenomena they suggest the number, material and/or form of solid bodies in PMCA batches as factors that may influence the efficacy of PrPres amplification. Consistent with this conclusion Gonzales-Montalban and colleagues [27] reported that the diameter of Teflon beads was of critical importance for the bead effect on PMCA.

From the results of our quantitative seeding activity assay we were able to correctly estimate, as validated by titrations in hamsters, the residual infectivity on test wires subjected to 15 different procedures or formulations exerting various mechanisms of action for prion disinfection. Reductions of scrapie titres ranged from less than ≤10^1^- to ≥10^5.5^-fold. In our PMCA assay we found a consistent quantitative correspondence between the biochemical seeding activity and biological infectivity of 263K scrapie prions. Additionally to this quantitative correlation we showed that PrP seeding activity present in scrapie brain homogenate or multiplied by PMCA could be transmitted to and propagated in the biological system of cerebral glial cell cultures from hamsters. Thus, PrP seeding was demonstrated in our experimental setup to represent both a biochemically and biologically active and replicative principle *in vitro*.

The results of our study have different methodological, conceptual and practical implications. Firstly, they show that PrP seeding activities can be quantitatively detected, and translated into scrapie infectivity titres, on steel wires used as model carriers for disinfection by an *in vitro* method that supplements the recently published approaches of qPMCA [9] and RT-QuIC [10]. However, other than qPMCA and RT-Quic our seeding activity assay is based on an internal calibration. Our test samples were subjected to PMCA always together with defined reference samples and processed in one sample set in the same sonicator. Yet, we occasionally observed differences in the Western blot staining intensities of similar PrPres reference standards that were independently blotted. Such inter-blot variation can be seen in Figure 4 when the signals in lanes R on the reference wires blots in sample sets 1 and 2 are compared to those in sample set 3. We noted that such variations occurred when different preparations of Western blot reagents were used, and it cannot be ruled out that blocking reagents, components of the chemoluminescence kit, PrPres reference standards and molecular mass standards for sample sets 1 and 2 on the one hand, and for sample set 3 on the other hand, were taken from different preparations. Although we intended to minimize such variations they could not be completely avoided due to the need for a regular renewal of depleted reagent stocks. Against this background, we did quantitatively compare Western blots only within internally calibrated sets of samples that had been processed together for PMCA and Western blotting (using the same preparations of blotting reagents), but not between independently processed sample sets. When doing so, neither variations in the efficacy of protein misfolding amplification (such as occasionally observed between sample sets processed in different sonicators, or between sample sets processed at differerent times in the same sonicator), or of Western blotting, nor subtle differences in the PrPres contents of different preparations of PrPres reference standards were found to bias the results of our seeding activity assay.

Consistent with the quantitative correlation between scrapie seeding activity and prion infectivity observed in our study, Wilham et al. [10] recently reported results that also pointed to a direct quantitative correspondence between the seeding and infectious activities of 263K scrapie prions measured by RT-QuIC and bioassay. This provides a remarkable concurrence given the methodological differences of the two studies, and seems to further substantiate the association between these prion-associated activities.

Secondly, we were able to establish a cell culture assay, using cerebral glial cells from hamsters, that showed accumulation of PrPres and amplification of proteinaceous seeding activity upon challenge with 263K scrapie brain homogenate or PMCA products derived from this prion strain. Wang et al. [33] recently reported the induction of PrPres accumulation in cultures of SN56 cells that had been exposed to recombinant murine PrPres produced by PMCA. In our study we show that glial cell cultures were not only susceptible to PrP seeding activity in terms of inducing PrPres accumulation but also capable of propagating seeding activity as evidenced by PMCA. This demonstrates scrapie seeding activity as a biologically active and replicative principle *in vitro*. So far, infection of cultivated cells with 263K scrapie prions was reported only in one publication [34]. Thus, our cell culture assay may provide a helpful novel tool in different areas of TSE research working with the frequently used 263K scrapie agent. Specifically, neither cell cultures susceptible to 263K scrapie-derived PMCA products, nor the direct demonstration, by PMCA, of the replication of prion seeding activity in cell cultures have been described, yet. Multiplied scrapie seeding activity derived by PMCA from 263K scrapie prions had been previously demonstrated to be associated with replicated infectivity *in vivo* [35], [36]. However, formally, such association cannot be taken for granted under experimental conditions other than exactly those examined in these (or similar) reports. For obvious reasons (e. g. ethical considerations or costs) the association between PrP seeding activity and prion infectivity can be validated only exemplarily in animals. In contrast, our cell culture assay may provide a practicable standard for the routine validation *in vitro* of PMCA-detected scrapie seeding activity as a biologically active and replicative principle.

Thirdly, when biochemical assays for the quantification of prion seeding activity are accompanied by a demonstration of the latter's propagation also in a biological system such as glial cell cultures, this seems to quantitatively and qualitatively mimic the *in vivo* detection of prion infectivity as exemplified in our experimental setup. Such approach may thus provide an effective or even superior alternative to bioassays in animals for various research purposes. Cell culture-coupled PMCA is faster, cheaper and ethically less critical than *in vivo* titrations of prion infectivity. In addition, it has a higher sensitivity and allows testing with a larger throughput. While the test limit of bioassays for the reduction of scrapie titres on steel wires was found to be about 5.5 logs in our [29] or other *in vivo* carrier assays for disinfection [21], [23], [25], at least 7 logs of seeding activity reduction can be monitored by PMCA testing of steel wires. Therefore, our *in vitro* approach may better resolve the safety margins of different prion disinfectants found to completely abolish detectable infectivity in bioassays. It may also scale the efficacy of disinfectants with higher differentiation than bioassays in hamsters, provided that reference wires contaminated with serially 2- or 5-fold- (instead of 10-fold-) diluted SBH were used for calibration.

Fourthly, in our experimental setup, we have shown that PrP seeding represents both a biochemically and biologically replicating and active principle *in vitro*, and that the former is quantitatively linked to prion infectivity titrated on steel wires *in vivo*. This would be compatible with PrP seeding and prion infectivity as corresponding manifestations of the same replicative activity as implicated by the nucleation-polymerization model [4] of prion replication.

However, with respect to the latter two points, additional studies are necessary to further substantiate the observed association between the seeding activity and biological infectivity of 263K prions for other disinfectant formulations and modes of inactivation. In addition, the methodological, conceptual and practical results described in this report for 263K scrapie prions should be validated for the most relevant human TSE agents [37], [38] (such as prions associated with sporadic CJD [sCJD]/subtypes MM1 or VV2 [39], or variant CJD) on different types of carriers or surfaces [40]. For this purpose, PMCA protocols and cell culture protocols need to be adapted for the propagation and detection of PrP seeding activities associated with sCJD or vCJD prions.

Finally, the findings obtained from our recent [22] and present work suggest that the development of broad-range disinfectants may substantially benefit from the use of prions as model pathogens for disinfection in the following four-stage procedure: Stage 1) Novel candidate formulations or procedures will be tested first in *in vitro*-assays that gauge the reduction of PrP^TSE^ as a static biochemical marker of prion infectivity. Stage 2) In a subsequent *in vitro* assay the decrease of PrP seeding activity will be measured as a dynamic marker for prion infectivity, and the reduction of prion infectivity will be estimated. The biological activity of PrP seeding assayed by PMCA may be validated in a complementary cell culture assay. Stage 3) Formulations or procedures found to be effective on prions in the screening at stages 1 & 2 are further tested in microbiological assays for their activities against bacteria, viruses and fungi. Stage 4) Formulations and procedures that successfully passed these *in vitro* tests shall be ultimately validated for the reduction of prion infectivity by *in vivo* assaying in animals. Advantageously, all tests of this staged procedure can be performed in solution or on test carriers, and with a largely reduced need for experiments in animals.

## Materials and Methods

### Ethics Statement

All animal work performed in this study was conducted under the European directive regarding the protection of animals used for experimental and other scientific purposes in strict accordance with the German Animal Welfare Act (Tierschutzgesetz) and adhering to the guidelines for the practical implementation of the German Animal Welfare Act published by the Charité – University Medicine Berlin (a joint institution of the Free University of Berlin and the Humboldt-University of Berlin, Germany). The protocol was reviewed and approved by the responsible Committee on the Ethics of Animal Experiments (“Tierversuchskommission – Berlin”) affiliated at the Authority for Animal Protection in Berlin (“Landesamt für Gesundheit und Soziales Berlin”, Berlin, Germany; http://www.lageso.berlin.de; Permit Number G0203/03). All surgery was performed under Ketavet/Rompun anesthesia, and all efforts were made to minimize suffering. According to German regulations, the sacrifice of normal Syrian hamsters for experimental purposes such as pursued in our study did not require approval by ethics committees or animal protection authorities. However, we voluntarily reported euthanasia of normal Syrian hamsters for the removal of brain tissue to the animal protection authority which registered this notification (Landesamt für Gesundheit und Soziales Berlin, Berlin, Germany; Registration Number T0220/07).

### Contamination, processing and seeding activity analysis of stainless steel wires used as model carriers for prion disinfection

The contamination and processing of stainless steel wires used as model carriers for disinfection was performed as described elsewhere [19] with modifications. In brief: Stainless steel wire (DIN-No. 1.4301, Forestadent, Pforzheim, Germany; diameter 0.25 mm) was cut into 5 mm long pieces. The resulting test bodies (here called wires) had a surface of ∼4.0 mm^2^, were washed in 2% Triton X-100 for 15 min under constant sonication (Sonorex RK 102 P; Bandelin Electronics), rinsed in distilled water, dried and sterilized in a steam autoclave at 121°C for 20 min. For contamination with scrapie prions, batches of 15 test steel wires were incubated in 150 ml of 10^−1^-diluted 263K scrapie hamster brain homogenate (SBH, containing 0.1 g of brain tissue per 1 ml) for 2 h under constant shaking at 37°C and 700 revolutions per minute (rpm) in a thermomixer (Amersham Biosciences). Batches of 15 reference steel wires each were similarly contaminated with 10^−1^-, 10^−2^-, 10^−3^-, 10^−4^-, 10^−5^-, 10^−6^-, 10^−7^- and 10^−8^-diluted SBH, and batches of 15 negative control steel wires were contaminated by incubation in 150 ml of 10^−1^-diluted normal hamster brain homogenate (NBH). 10^−1^-dilutions of SBH and NBH were prepared in Tris-buffered saline (TBS: 10 mM Tris-HCl, 133 mM NaCl, pH 7.4). However, lower concentrated SBH was prepared by dilution in 10% (w/v) NBH in order to warrant a comparable load of biological contamination with normal hamster brain tissue on test-, reference- and negative control steel wires.

Following removal of the scrapie- or normal hamster brain homogenates, wires were transferred to and placed separately from each other in Petri dishes, air-dried for 1 h, and stored overnight (∼16 h) at room temperature. Dried reference and control steel wires were recollected in 0.5 ml Eppendorf safe lock tubes. Batches of 15 contaminated test steel wires were exposed to the following formulations or procedures for prion disinfection:

(a) 1.0 M sodium hydroxide (NaOH, 60 min, 23°C); (b) 2.5% (w/v) sodium hypochlorite (NaOCl, >20 000 ppm available chlorine, 60 min, 23°C); (c) 4.0 M guanidine thiocyanate (GdnSCN, 10 min, 23°C); (d, e) a mixture of 0.2% (w/v) SDS and 0.3% (w/v) sodium hydroxide (in the following referred to as 0.2% SDS/0.3% NaOH) applied for 5 min (d) or 10 min (e) at 23°C; (f) 0.2% SDS/0.3% NaOH in 20% (v/v) n-propanol (10 min, 23°C); (g–i) an alkaline cleaner for medical devices [41] at concentrations of 1.0% (v/v) applied for 60 min at 23°C (g) or 0.5% (v/v) applied for 5 min (h) or 10 min (i) at 55°C; (j) 5% (w/v) sodium dodecylsulfate (SDS, 60 min, 90°C); (k) 2% (v/v) glutardialdehyde (adjusted to a neutral pH of 7.0 with 0.1 M phosphate buffer, 10 min, 23°C); (l) 2% (v/v) glutardialdehyde (non buffered, pH 4.6), 10 min, 23°C); (m) Cidex OPA (containing 0.55% (v/v) *ortho*-phthalaldehyde, 10 min, 23°C); (n) 0.25% (v/v) peracetic acid (60 min, 23°C; (o) porous load steam sterilization at 134°C and 3 bar for 5 min (performed on wires sealed in sterilization foil).

Test wires were incubated in 1.5 ml of the disinfectant formulations in a thermomixer (400 rpm) at the temperatures and for the times specified above, or subjected to steam sterilization at 134°C. Finally, all test wire batches were rinsed under constant shaking five times, each time in 45 ml distilled water for 10 min at room temperature. After rinsing in distilled water, processing of test wires was finished by air-drying in Petri dishes for 1 h, storing overnight (∼16 h) at room temperature, and recollection of batches in 0.5 ml Eppendorf safe lock tubes.

The scrapie seeding activities of test-, reference- and negative control steel wires were quantitatively assessed per batch by PMCA and densitometric Western blotting.

### Protein misfolding cyclic amplification

The PMCA procedure described in the following was established based on previously published protocols [5], [7], [8], [31], [36], [42], [43] with 263K scrapie brain homogenate as seeding material, and subsequently applied to test-, reference- and negative control steel wires of the carrier assay for prion disinfection.

#### Preparation of amplification substrate

Adult normal Syrian hamsters were euthanized by exposure to CO_2_ and transcardially perfused with 5 mM EDTA/phosphate buffered saline (PBS, 8 mM Na_2_HPO_4_, 1.5 mM KH_2_PO_4_, 137 mM NaCl, 2.7 mM KCl, pH 7.4). Hamster brains were removed and homogenized in ice-cold conversion buffer (PBS containing complete protease inhibitor cocktail [Roche], 4 mM EDTA and 1% Triton-X-100). The homogenate was adjusted to a concentration of 1 g brain tissue per 10 ml (i. e. to a 10^−1^-dilution, or 10% [w/v]) and subjected to centrifugation in a Heraeus Varifuge 3.0 at 3000 rpm and 4°C for 5 min. The clarified supernatant was collected and stored as NBH-PMCA substrate in aliquots at −70°C until use.

#### Amplification of PrP misfolding

In order to prevent cross-contamination of samples during PMCA all pipetting steps were performed with stuffed single-use pipette tips, and gloves were changed each time before handling a new sample. Serial PMCA was performed in 0.5 ml Eppendorf safe lock tubes that had been subjected to steam sterilization at 121°C and 3 bar for 20 min prior to use. Approximately 30 µl of glass beads (diameter 0.5–0.75 mm; Roth, Germany) were filled into each reaction tube. The glass beads had been both washed five times with distilled water and subsequently subjected to steam sterilization at 121°C and 3 bar for 20 min. Using reaction tubes prepared in this way we performed four sets of PMCA experiments:

(i) 10 µl-aliquots of 10^−1^-diluted NBH containing 10^−8^, 10^−9^, 10^−10^, 10^11^, 10^−12^ or 0 g of homogenized 263K scrapie brain tissue were mixed in the reaction tubes with 140 µl of NBH-PMCA substrate for PMCA experiments without steel wires. (ii) Similar mixtures containing 10^−9^, 10^−10^ or 0 g of homogenized 263K scrapie brain tissue in NBH-PMCA substrate were used for PMCA in the presence, in each PMCA round, of 15 steel wires, or without steel wires, in order to test the influence of steel wires on the efficacy of PMCA. (iii) For PMCA experiments with test-, reference- and negative control steel wires of the carrier assay for prion disinfection batches of 15 wires each were added to 150 µl of NBH-PMCA substrate. (iv) Finally, 5 and 10 µl aliquots of extracts from hamster glial cell cultures were subjected to PMCA in 145 µl and 140 µl NBH-PMCA substrate, respectively, for validating cell-mediated propagation of scrapie seeding activity.

The reaction tubes from all sets of experiments were sealed with parafilm and paraffin wax, and disinfected in 0.2% SDS/0.3% NaOH for 5 min [29] after sealing. Serial PMCA was carried out using an automatic ultrasonification apparatus (Sonicator 3000MPD or Sonicator 4000MPX from Misonix, New York, USA). One round of PMCA consisted of 24 alternating cycles of ultrasonification (180–190 W for 40 sec every 60 min) and incubation of samples at 37°C between ultrasonifications. The reaction vials were placed in 4 M GdnSCN solution [44] above the ultrasonic transducer, and after completion of a PMCA round the sealed reaction tubes were disinfected once more in 0.2% SDS/0.3% NaOH for 5 min. Subsequently, reaction mixtures were gently spun down and collected by vial puncture using disposable hydrodermic 20 gauge needles. From the harvested reaction mixtures 30 µl aliquots were mixed with 120 µl of fresh NBH-PMCA substrate in new safe lock tubes containing glass beads. For the next round of PMCA the vials were sealed and disinfected again as described above.

Sets of PMCA experiments without steel wires (i and iv) comprised three rounds of amplification, while those with steel wires (ii and iii) comprised four rounds. 30 µl aliquots from PMCA-batches collected prior to PMCA or obtained after 1, 2, 3 or 4 rounds of PMCA were mixed with 3 µl 13% (w/v) sarcosyl, 0.6 µl 4% (w/v) SDS and 6 µl of Proteinase K stock solution (1 mg PK/ml; final concentration of PK in digest samples: 150 µg/ml), incubated for 1 h at 55°C, and centrifuged at 13.000 rpm and ambient temperature for 1 min (Heraeus Biofuge pico). 30 µl aliquots of the supernatant were mixed with sample loading buffer (125 mM Tris pH 6.8, 20% glycerol, 10% mercaptoethanol, 4% SDS, 0.05 % [w/v] bromphenol blue) and incubated for 10 min at 99°C. After addition of phenylmethylsulfonyl fluoride (PMSF) to a final concentration of 2.5 mM 10 µl aliquots of the samples were subjected to SDS-PAGE and Western blotting for the detection of PK-resistant prion protein.

### SDS-PAGE and Western blotting

SDS-PAGE and Western blotting using the monoclonal anti-PrP antibody 3F4 [45] for the detection of PrP were performed as described elsewhere [28]. Marker proteins of 30- and 20 kDa (Pharmacia, Germany) were used to specify the molecular mass of blotted PrP. PK-digested homogenate from 263K scrapie hamster brains used as an internal PrPres reference standard in Western blot analyses was prepared, from different donor animals, as outlined previously [28], [46].

### Densitometric Western blot analysis

Western blots were scanned in transmission mode and electronically stored as bitmap (BMP) files. Densitometry was performed using the open source software *Image J* (http://rsbweb.nih.gov/ij/). For densitometric measurements the image type was set to “8-bit” and blot pictures were inverted. Rectangles were used to circumscribe the Western blot signals for monomeric un-, mono- and diglycosylated PrPres in individual Western blot lanes within one frame, and integrated signal densities were recorded. Unspecific backgound staining on the blot membrane was collected for similarly large areas and subtracted from the corresponding PrPres measurements. Relative intensities of PrPres signals were calculated as a percentage of the signal intensities of PrPres reference standards (i. e. the integrated signal densities of samples were divided by the integrated signal densities of PrPres reference samples on the same Western blot after correction for the background).

Relative intensities of PrPres signals after 1, 2, 3 or 4 rounds of PMCA were plotted, using the software *SigmaPlot 11.0* (Systat Software, Germany), for PMCA samples seeded by i) different amounts of homogenized 263K scrapie brain tissue, ii) reference steel wires contaminated with 10^−1^-, 10^−2^-, 10^−3^-, 10^−4^-, 10^−5^-, 10^−6^-, 10^−7^- and 10^−8^-diluted SBH, or iii) test steel wires exposed to different formulations/procedures for disinfection after contamination with 10^−1^-diluted SBH. Upper parts of symmetrical error bars were reproduced in plots in order to indicate the variation (i. e. the range) of results for duplicate reference samples and individual test samples after three independently performed densitometric measurements of Western blot signals.

Residual seeding activities on test wires (SA_TW_) were quantitatively assessed based on a systematic comparison of the levels of PrPres amplification measured for individual batches of test wires and duplicate batches of reference wires after PMCA. Maximum-, mean- and minimum values of the seeding activity of test wires (SA_TW[Max.]_, SA_TW[Mean]_, SA_TW[Min.]_) were assessed for 1, 2, 3 and 4 rounds of PMCA by comparing the upper range, the mean, and the lower range of measured PrPres staining seeded by test wires to the lower range, the mean, and the upper range of measured PrPres staining seeded by reference wires, respectively. The scrapie seeding activities of reference wires contaminated with 10^−1^-, 10^−2^-, 10^−3^-, 10^−4^-, 10^−5^-, 10^−6^-, 10^−7^- and 10^−8^-diluted SBH were referred to as SA_RW_(10^−1^), SA_RW_(10^−2^), SA_RW_(10^−3^), SA_RW_(10^−4^), SA_RW_(10^−5^), SA_RW_(10^−6^), SA_RW_(10^−7^) and SA_RW_(10^−8^).

Furthermore, we observed the following specific criteria in our densitometric analyses: i) Test and reference samples showing relative staining intensities that did not differ for more than 10 percentage points were considered as approximately equal, since the overall variation found for reference samples in our study was 5±5 (mean ± SD) percentage points (not shown). ii) When the relative intensities of those reference samples that were in the saturation region of Western blot staining did not exceed the relative staining intensities of test samples for at least 10 percentage points, they were not used for an assessment. iii) If no signal could be detected for a test sample at a specific round of PMCA such finding was used for an assessment of the lowest reference seeding activity larger than that of the test wires, only (provided that the relative intensity of a relevant reference signal was at least 10%).

Finally, consolidated values of the residual seeding activities on test wires (SA_TW[Cons.]_) were deduced from the individual assessments of SA_TW[Max.]_, SA_TW[Mean]_ and SA_TW[Min.]_ for 1, 2, 3 and 4 rounds of PMCA.

### Assessment and bioassay validation of prion infectivity on test wires of the carrier assay for disinfection

The levels of infectivity present on reference wires, negative control wires and on test wires exposed to formulations/procedures (a), (b), (d), (e), (f), (g), (h), (i), (j) or (o) had been determined previously [22], [29], while the infectivity levels remaining attached to test wires processed as decribed above for formulations (c), (k), (l) or (m) were determined in this study by bioassay in hamsters as described elsewhere using a dose-response relationship established by end-point-titration [29].

The known infectivity titres of steel wires identical to those used as reference wires in the seeding activity assay of this study were applied as conversion factors for a tentative translation of the seeding activities detected on test wires into titre estimates of biological prion infectivity. These titre estimates were then validated by comparison to the actual infectivity levels on the test wires found in hamster bioassays.

### Glial cell culture assay for scrapie seeding activity

#### Preparation of glial cell cultures from hamsters

Primary glial cell cultures were prepared based on the protocol by Lima et al. [47] with modifications: Neonatal Syrian hamsters were sacrificed 2–3 days after birth by decapitation. Brains were dissected and stored in ice cold PBS/glucose (1000 mg/l D-glucose, 100 U/ml penicillin, 100 µg/ml streptomycin and 0.5 µg/ml partricin) until further processing. The cerebellum and meninges were removed, and the remaining tissue was minced using a sterile razor blade. The minced tissue was washed in PBS/glucose and centrifuged for 3 min and 1000 rpm (Heraeus Varifuge 3.0) at ambient temperature. The supernatant was discarded, and the pellet was washed again in PBS/glucose prior to a second centrifugation as described. The pellet was resuspended in 5 ml Dulbecco's modified Eagle medium [DMEM] containing 10% [v/v] fetal calf serum [FCS], 1000 mg/l D-glucose, 3 mM sodiumbicarbonate, 2 mM L-glutamine, 100 U/ml penicillin, 100 µg/ml streptomycin and 0.5 µg/ml partricin, and dispersed by gentle trituration. A cell strainer (pore size: 40 µm, Becton Dickinson) was used for the effective separation of cells and the preparation of a homogeneous single-cell-suspension. The cell suspension was centrifuged (5 min at 1200 rpm, ambient temperature, Heraeus Varifuge 3.0) and resuspended in 10 ml growth medium (GM: DMEM containing 10% [v/v] FCS, 1000 mg/l D-glucose, 2 mM L-glutamine, 100 U/ml penicillin, 100 µg/ml streptomycin, 0.5 µg/ml partricin]. The cell density was determined by vital staining with trypan blue. 3×10^6^ cells were grown in 25 ml GM using plastic cell culture flasks (175 cm^2^, NUNC) that had been pre-coated with poly-L-lysin (1 mg/ml). Cultures were incubated at 37°C in a humidified 5% CO_2_–95% air atmosphere. Cell debris were removed by washing with PBS, GM (25 ml) was renewed, and cells were cultivated for a further seven days. Subsequently, for passage, cultures were washed with PBS and incubated with 0.05% trypsin in PBS until cells were completely detached from the flask and separated from each other (Note: Enzymatic detachment and separation of cells are of crucial importance for the susceptibility to PrP seeding). Cells were centrifuged (5 min at 1200 rpm, ambient temperature, Heraeus Variofuge 3.0), resuspended in GM and strained (cell strainer: pore size 40 µm). The cell density was determined by vital staining with trypan blue, and 1,25×10^5^ cells were cultured for two days in 12.5 ml GM using plastic cell culture flasks (75 cm^2^, NUNC) that had been pre-coated with poly-L-lysin (1 mg/ml). Cultures were incubated at 37°C as described above.

#### Infection and harvesting of glial cell cultures

Glial cell cultures adjusted to a cell density 1,25×10^5^ per flask were exposed to i) 10^−1^-diluted NBH containing 0 g, 2.5×10^−5^ g or 1.0×10^−6^ g of 263K scrapie brain tissue per challenged culture, or ii) to concentrated PMCA products seeded by negative control wires (coated with 10^−1^-diluted NBH), reference wires (contaminated with 10^−8^-diluted SBH), or test wires (contaminated with 10^−1^-diluted SBH) that had been treated for disinfection by formulations/procedures (a), (c) or (j). 80 µl of harvested PMCA products were centrifuged at 45.000 rpm and 4°C for 2.5 h in an Optima-max-ultracentrifuge (Beckman) using a TLA-45 rotor, and the pellet was resuspended in 150 µl PBS. 10 µl of the suspensions containing the inocula according to i) or ii) were added to cell cultures in 12.5 ml GM. After three days of cultivation as described above the inoculum was removed, and the cells were washed once with PBS.

Immediately after removal of the inoculum and washing of the cells (i.e. at 3 days post initial exposure [DPE]) baseline cultures were harvested, while in the other cultures the culture medium was renewed and subsequently changed once a week until harvesting of cells at 20, 31, 40 or 60 DPE. Cells were harvested by lysis in 800 µl 1% (w/v) sarcosyl and mechanical detachment using a cell scraper. The resulting cell solutions were collected and centrifuged at 45.000 rpm and 4°C for 2.5 h in an Optima-max-ultracentrifuge (Beckman) using a TLA-45 rotor. The resulting pellets were resuspended in 100 µl PBS.

#### Analysis of glial cell cultures for PrP^C^ expression and PrPres accumulation

For the testing of PrP^C^ expression 5 µl of resuspended pellets from cell cultures that had been exposed to 10^−1^-diluted NBH and harvested at 40 DPE were incubated with 45 µl of sample loading buffer for 10 min at 99°C, and 10 µl aliquots were subjected to SDS-PAGE and PrP-Western blotting.

For the detection of PrPres accumulation 50 µl of resuspended cell culture pellets (see above) were mixed with 5 µl 13% (w/v) sarcosyl and 10 µl of Proteinase K stock solution (1 mg PK/ml; final concentration of PK in digest samples: 150 µg/ml) and incubated for 1 h at 37°C. Subsequently samples were mixed with an equal volume of sample loading buffer and incubated for 10 min at 99°C. After addition of phenylmethylsulfonyl fluoride (PMSF) to a final concentration of 2.5 mM 10 µl aliquots of the samples were subjected to SDS-PAGE and Western blotting for the detection of PK-resistant prion protein.

#### Analysis of glial cell cultures for propagation of seeding activity

5 µl aliquots of undiluted cell extracts harvested at 3 DPE and 40 DPE after challenge of cultures with 10^−6^ g of scrapie brain tissue, NBH, or a PMCA product that had been seeded by test wires treated for disinfection with formulation (a) were subjected to PMCA for the detection of seeding activity. In a similar experiment with cultures harvested after challenge with a PMCA product that had been derived from test wires treated for disinfection with formulation (c) 5 µl of a 1:25-diluted cell extract were used for PMCA seeding. Detection of PrPres in 30 µl aliquots from PMCA-batches obtained after 1, 2 or 3 rounds of PMCA by SDS-PAGE and Western blotting was performed as described above.

## Supporting Information

Figure S1 **Expression of PrP^C^ in glial cell cultures.** Western blot detection of PrP^C^ in a glial cell culture harvested at 40 days post initial exposure to 10^-1^-diluted NBH. Lanes 1, 2 and 3 represent 1.0, 0.1 and 0.01 μl-aliquots from resuspended cell culture pellets, respectively. Lane M, molecular mass marker (30 kDa).
(TIF)

Table S1 **Hamster bioassay with test wires subjected to different formulations for disinfection.**
(DOC)

Table S2 **Residual infectivity and titre reductions on test wires subjected to different formulations for disinfection.**
(DOC)
