# Supplementary figures and images for: Quantitative Detection and Biological Propagation of Scrapie Seeding Activity In Vitro Facilitate Use of Prions as Model Pathogens for Disinfection
Source: PLoS One. 2011 May 27;6(5):e20384. doi: 10.1371/journal.pone.0020384 (PMC3103549; doi:10.1371/journal.pone.0020384)

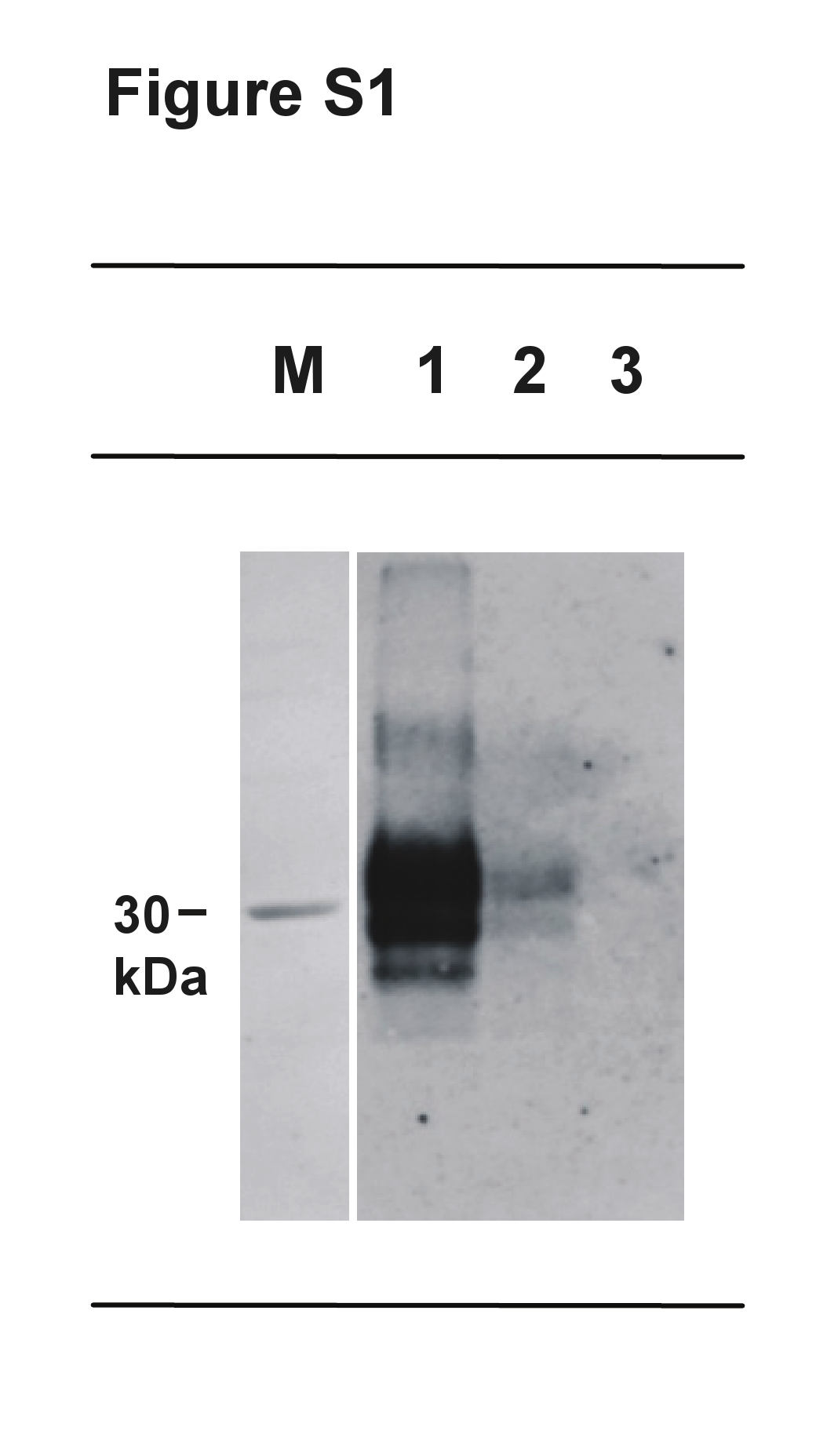

Supplement: Figure S1 — Expression of PrPC in glial cell cultures. Western blot detection of PrPC in a glial cell culture harvested at 40 days post initial exposure to 10-1-diluted NBH. Lanes 1, 2 and 3 represent 1.0, 0.1 and 0.01 μl-aliquots from resuspended cell culture pellets, respectively. Lane M, molecular mass marker (30 kDa). (TIF) [file pone.0020384.s001.tif]
