# Supplementary material for: Quantitative Detection and Biological Propagation of Scrapie Seeding Activity In Vitro Facilitate Use of Prions as Model Pathogens for Disinfection
Source: PLoS One. 2011 May 27;6(5):e20384. doi: 10.1371/journal.pone.0020384 (PMC3103549; doi:10.1371/journal.pone.0020384)
Supplement: Table S1 — Hamster bioassay with test wires subjected to different formulations for disinfection. (DOC) [file pone.0020384.s002.doc]

| **Table S1. Hamster bioassay with test wires subjected to different formulations for disinfection** | | | | | | | |  |
| --- | --- | --- | --- | --- | --- | --- | --- | --- |
|  |  |  |  |  |  |  |  |  |
|  |  |  |  |  |  |  |  |  |
|  |  |  |  | **Bioassay group 1** | |  | **Bioassay group 2** | |
|  |  |  |  |  |  |  |  |  |
|  |  |  |  |  |  |  |  |  |
| **Formulation** | **Concen-** | **Time** | **Tempera-** | **Attack** | **Survival time** |  | **Attack** | **Survival time** |
|  | **tration** | **[min]** | **ture [°C]** | **rate** | **[dpim]** |  | **rate** | **[dpim]** |
|  |  |  |  |  |  |  |  |  |
|  |  |  |  |  |  |  |  |  |
| **GdnSCN** | **4.0 M** | **10** | **23** | **0/5*** | ***500*** |  | **n.d.** | **n.d.** |
|  |  |  |  |  |  |  |  |  |
| **Glutardialdehyde** | **2.0 %** | **10** | **23** | **6/6** | **112±8** |  | **6/6** | **112±4** |
| **(neutralised, pH 7.0)** |  |  |  |  |  |  |  |  |
|  |  |  |  |  |  |  |  |  |
| **Glutardialdehyde** | **2 %** | **10** | **23** | **6/6** | **104±3** |  | **6/6** | **107±6** |
| **(non neutralised, pH 4.6)** |  |  |  |  |  |  |  |  |
|  |  |  |  |  |  |  |  |  |
| **Cidex OPA** | **0.55 %** | **10** | **23** | **3/3** | **104±2** |  | **3/3** | **107±5** |
|  |  |  |  |  |  |  |  |  |
|  |  |  |  |  |  |  |  |  |

**Footnote to Table S1:** For contamination, test wires were incubated in 150 μl of 10^-1^-diluted 263K brain homogenate which achieved an initial load of infectivity of about 3x10^5^ 50% lethal doses of 263K scrapie infectivity following intracerebral implantation (LD_50i.c.imp_) per wire [29]. Attack rates for scrapie are specified as number of animals that developed terminal symptoms per total number of challenged animals. Survival times until the development of terminal scrapie are provided in days post implantation (dpim; means ± SD), and survival times provided in *italics* refer to hamsters that were sacrificed at the indicated time points without having developed clinical symptoms of scrapie. Explanation of symbols: ^*^ One out of six animals challenged with an implanted wire died for reasons unrelated to scrapie (accordingly, the number of animals in the respective group was set to n=5). Explanation of abbreviations: n.d. – not determined.
