# Supplementary material for: Quantitative Detection and Biological Propagation of Scrapie Seeding Activity In Vitro Facilitate Use of Prions as Model Pathogens for Disinfection
Source: PLoS One. 2011 May 27;6(5):e20384. doi: 10.1371/journal.pone.0020384 (PMC3103549; doi:10.1371/journal.pone.0020384)
Supplement: Table S2 — Residual infectivity and titre reductions on test wires subjected to different formulations for disinfection. (DOC) [file pone.0020384.s003.doc]

| **Table S2. Residual infectivity and titre reductions on test wires subjected to different formulations for disinfection** | | | | | | | | |
| --- | --- | --- | --- | --- | --- | --- | --- | --- |
|  |  |  |  |  |  |  |  |  |
|  |  |  |  |  |  |  |  |  |
|  |  |  |  | **Bioassay group 1** | |  | **Bioassay group 2** | |
|  |  |  |  |  | |  |  | |
|  |  |  |  |  |  |  |  |  |
| **Formulation** | **Concen-** | **Time** | **Tempera-** | **Res. Inf./Wire** | **Red.** |  | **Res. Inf./Wire** | **Red.** |
|  | **tration** | **[min]** | **ture [°C]** | **[LD_50i.c.imp_]** | **[logs_10_]** |  | **[LD_50i.c.imp_]** | **[logs_10_]** |
|  |  |  |  |  |  |  |  |  |
|  |  |  |  |  |  |  |  |  |
| **GdnSCN** | **4.0 M** | **10** | **23** | **UD** | **≥ 5.5** |  | **n.d.** | **n.d.** |
|  |  |  |  |  |  |  |  |  |
| **Glutardialdehyde** | **2.0 %** | **10** | **23** | **≈ 3 x 10^3^** | **≈ 2** |  | **≈ 3 x 10^3^** | **≈ 2** |
| **(neutralised, pH 7.0)** |  |  |  |  |  |  |  |  |
|  |  |  |  |  |  |  |  |  |
| **Glutardialdehyde** | **2.0 %** | **10** | **23** | **> 3 x 10^3^** | **> 1** |  | **> 3 x 10^3^** | **> 1** |
| **(non neutralised, pH 4.6)** |  |  |  | **to < 3 x 10^4^** | **to < 2** |  | **to < 3 x 10^4^** | **to < 2** |
|  |  |  |  |  |  |  |  |  |
| **Cidex OPA** | **0.55 %** | **10** | **23** | **> 3 x 10^3^** | **> 1** |  | **> 3 x 10^3^** | **> 1** |
|  |  |  |  | **to < 3 x 10^4^** | **to < 2** |  | **to < 3 x 10^4^** | **to < 2** |
|  |  |  |  |  |  |  |  |  |

**Footnote to Table S2:** Residual prion infectivity on test wires was deduced from attack rates and survival times (Table S1) by using a dose-response relationship previously established for steel wire bioassay in hamsters [29], and titre reductions were calculated by comparing the residual infectivity with the load of contamination prior to disinfection. Reduction of prion infectivity on test wires is expressed in log_10_ units. Explanation of abbreviations: n.d. – not determined; UD – undetectable; Red. - reduction of titre; Res. Inf. – residual infectivity.
